# An Increased Burden of Highly Active Retrotransposition Competent L1s Is Associated with Parkinson’s Disease Risk and Progression in the PPMI Cohort

**DOI:** 10.3390/ijms21186562

**Published:** 2020-09-08

**Authors:** Abigail L. Pfaff, Vivien J. Bubb, John P. Quinn, Sulev Koks

**Affiliations:** 1 Perron Institute for Neurological and Translational Science, Perth, WA 6009, Australia; abigail.pfaff@uwa.edu.au; 2 Centre for Molecular Medicine and Innovative Therapeutics, Murdoch University, Perth, WA 6150, Australia; 3 Department of Pharmacology and Therapeutics, Institute of Systems, Molecular and Integrative Biology, University of Liverpool, Liverpool L69 3BX, UK; jillbubb@liverpool.ac.uk (V.J.B.); jquinn@liverpool.ac.uk (J.P.Q.)

**Keywords:** Parkinson’s disease, retrotransposon, L1, retrotransposition competent

## Abstract

Long interspersed element-1 (LINE-1/L1s) contributes 17% of the human genome with more than 1 million elements present; however, fewer than 100 of these have evidence for being retrotransposition competent (RC). In addition to those RC-L1s present in the reference genome, there are a small number of known non-reference L1 insertions that are also retrotransposition competent. L1 activity, whether through the potentially detrimental effects of their mRNA or protein expression or somatic retrotransposition events, has been linked to several neurological conditions. The polymorphic nature of both reference and non-reference RC-L1s in terms of their presence or absence will result in individuals harboring a different combination of these elements and it is currently unknown if this type of germline variation contributes to the risk of neurological disease. Here, we utilized whole-genome sequencing data from 178 healthy controls and 372 Parkinson’s disease (PD) subjects from the Parkinson’s Progression Markers Initiative (PPMI) to investigate the role of RC-L1s in PD. In the PPMI cohort, we identified 22 reference and 50 non-reference polymorphic RC-L1 loci. Focusing on 16 highly active RC-L1 loci, an increased burden of these elements (≥9) was associated with PD (OR 1.25, 95% CI 1.03–1.51, *p* = 0.02). In addition, we identified significant associations of progression markers of PD and the burden of highly active RC-L1s. This study has identified a novel type of genetic element associated with PD risk and disease progression.

## 1. Introduction

**Figure 1:**
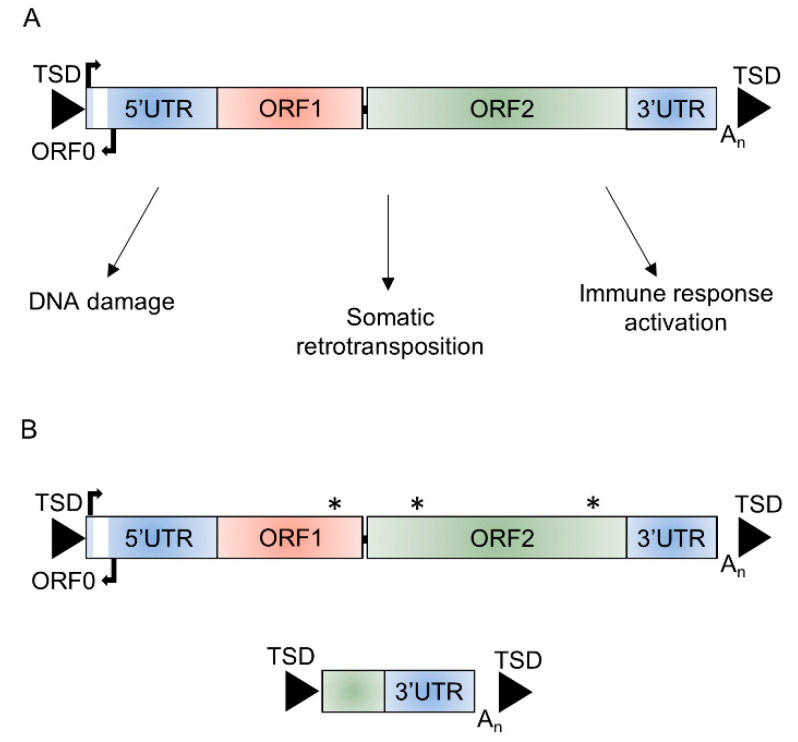
Structure of long interspersed element-1 (L1). (**A**) Schematic of a functional full-length L1 ~6 kb in length that consists of a 5′ untranslated region (5′UTR) containing the endogenous L1 promoter and an antisense promoter, three open reading frames (ORF0, ORF1, and ORF2), and a 3′ untranslated region (3′UTR) flanked by target site duplications (TSDs) and potential mechanisms through which retrotransposition-competent L1s could affect cellular function. (**B**) The majority (>99%) of L1 insertions are unable to retrotranspose due to inactivating or truncating mutations in ORF1 or ORF2, 5′ truncations, or inversions. * mutation affecting function of ORF encoded proteins.

Long interspersed element-1s (LINE-1/L1s) are the only autonomous retrotransposons in the human genome that contain elements currently able to mobilize. Although L1s contribute to 17% of the human genome with more than 1 million elements, there are only 146 full-length L1s with intact reading frames identified in the most recent version of the reference genome, hg38 [1]. An intact full-length L1 is ~6 kb in length; it consists of a 5′ untranslated region (UTR), three open reading frames (ORF0, ORF1, and ORF2), a 3′UTR, and poly A-tail and is usually flanked by target site duplications (TSD) generated upon insertion through target-primed reverse transcription (Figure 1). The proteins encoded by ORF1, a RNA binding protein, and ORF2, with endonuclease and reverse transcriptase activity, are required for L1 retrotransposition, which occurs through a ‘copy and paste’ mechanism [2,3,4,5,6]. The rate of de novo L1 insertions in the germline is estimated at 1/63 live births [7] and 124 L1 mediated retrotransposition events have been associated with genetic diseases [8]. Somatic retrotransposition is also known to occur in tumors and in the adult brain, with the rate of retrotransposition estimated at between 0.04 and 13.7 events per neuron [9,10]. Cellular assays have demonstrated the ability of L1s to retrotranspose in neural progenitor cells and in mature non-dividing neurons [11,12]. Differential expression of retrotransposon mRNA and proteins, including those of L1s, has also been identified in several different neurological conditions, including Rett syndrome, amyotrophic lateral sclerosis, Alzheimer’s disease, ataxia telangiectasia, and Aicardi–Goutieres syndrome [11,13,14,15,16,17,18]. L1s have also been shown to be activated in cellular and mouse models of Parkinson’s disease (PD) [19,20]. Derepression of L1s in senescent cells and aged mice has been shown to activate age-associated inflammation, suggesting a potential role for these elements in age-associated disorders, which includes neurodegenerative diseases [21].

The majority of L1 elements are inactive in terms of their ability to mobilize, with only an estimated 80–100 retrotransposition-competent L1s (RC-L1s) in the human genome (Figure 1) [22]. Both reference and non-reference L1 loci have been identified as retrotransposition competent [22,23,24]. Many of these RC-L1s are polymorphic for their presence or absence; therefore, the number and combination of these elements will differ between individuals [22,25,26]. We hypothesize that RC-L1s are involved in processes that lead to or exacerbate the pathological changes of certain neurological conditions and that polymorphic RC-L1s or their accumulation across the genome could predispose an individual to disease. Our study focused on PD, the second most common neurodegenerative disease, which is characterized by the loss of dopaminergic neurons from the substantia nigra and results in both motor and non-motor symptoms [27]. To investigate the genetic burden of RC-L1s in PD, we obtained whole-genome sequencing (WGS) and longitudinal phenotypic and clinical data from the Parkinson’s Progression Markers Initiative (PPMI). The PPMI has an extensive dataset to facilitate PD research, with the aim of understanding the processes leading to PD and the development of novel therapeutics [28]. The WGS enabled calling of RC-L1 loci genotypes across the genome of 372 PD subjects and 178 healthy controls to identify those RC-L1s that were polymorphic for their presence or absence. This data was integrated with the phenotypic and clinical data available from the PPMI subjects to determine the effects of the RC-L1s on the risk of developing PD and the progression of the disease.

## 2. Results

### 2.1. Characterizing Reference and Non-Reference RC-L1s

**Figure 2:**
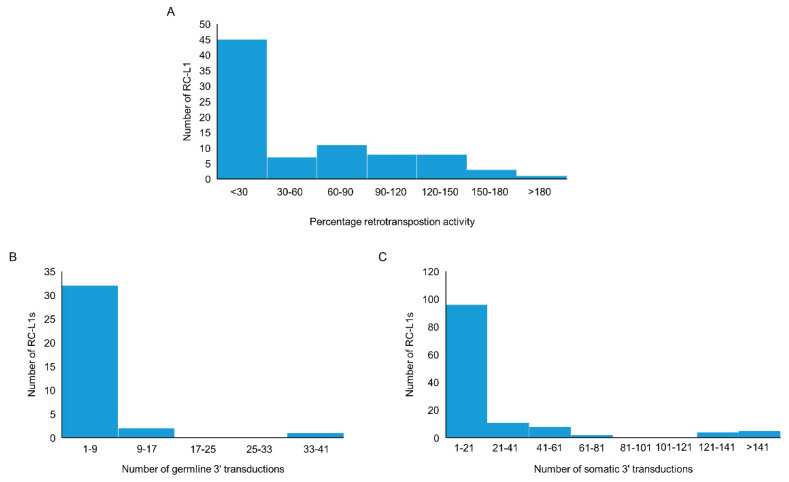
Characteristics of reference and non-reference retrotransposition competent (RC) L1s. (**A**) The distribution by percentage activity of 37 reference and 46 non-reference RC-L1s that demonstrated activity in a cellular retrotransposition assay. (**B**) The distribution of 23 reference and 13 non-reference RC-L1s by the number of germline insertions attributed to the specific RC-L1 using 3′ transduction events. (**C**) The distribution of 41 reference and 85 non-reference RC-L1s by the number of somatic insertions attributed to the specific RC-L1 using 3′ transduction events.

We compiled a list of RC-L1s based on published data. The criteria used included those L1s that have the ability to retrotranspose as evidenced by activity in a cellular retrotransposition activity, or those that were the source element for germline or somatic L1 insertions traced by 3′ transduction events [22,23,24,25,29,30]. Using this definition, 81 reference and 117 non-reference L1s were identified as retrotransposition competent (Tables S1 and S2). The cellular retrotransposition assay was used to benchmark the activity of L1s against an L1 element (L1_RP_ or L1.3) known to have a high level of retrotransposition activity. Of the 198 RC-L1s in our list, 83 had demonstrated the ability to retrotranspose in the cellular assay [22,23,24]. More than half of the RC-L1s (54.2%) had retrotransposition activity below 30% of the L1_RP_ or L1.3 and 23.9% had higher activity than the L1_RP_ or L1.3 (>100%) (Figure 2A). During L1 retrotransposition, 3′ transductions can occur when RNA polymerase II bypasses the canonical polyadenylation signal of the L1 element, resulting in part of the downstream genomic sequence being integrated into the genome along with the new L1 insertion. This can be used to identify the source element for L1 insertions and provide evidence for those elements that have to the ability to retrotranspose. From the list of 198 RC-L1s, 36 were identified as source elements generating germline L1 insertions; however, the majority gave rise to fewer than 9 insertions across the population studied (Figure 2B) [25]. There were 126 RC-L1s that had been identified as source elements for somatic L1 insertions in tumors and the majority gave rise to fewer than 21 insertions (Figure 2C) [29,30]. One reference RC-L1 located at chr22q12.1 was responsible for >900 somatic insertions in data combined from Tubio et al. and Rodriguez-Martin et al., which is nearly 3× greater than the RC-L1 responsible for the next highest number of somatic tumor insertions. The graphs in Figure 2 show that there were only a handful of elements at the higher end of the three defining parameters used for characterization of the RC-L1s and even fewer RC-L1s from our list that show the highest levels of retrotransposition activity based on all three parameters (Tables S1 and S2).

### 2.2. An Increase in the Number of Highly Active RC-L1s Is Associated with an Increased Risk of Parkinson’s Disease

In the PPMI cohort, 22 of the 81 reference and 50 of the 117 non-reference RC-L1s from our compiled lists were identified as polymorphic for their presence or absence. The other 59 reference RC-L1s that were not found to be polymorphic are likely to be fixed in the population as exemplified by insertion allele frequencies of 1 for several of these elements in published datasets, which includes the RC-L1 at chr22q12.1, which is responsible for highest number of somatic L1 retrotransposition events in cancer [22,25]. The non-reference RC-L1s not being detected in our cohort may be due to their low prevalence or absence in the Caucasian population analyzed here. The demographics of the PPMI cohort are shown in Table 1 and individuals all reported race as white in the cohort analyzed. Association analysis of the 72 polymorphic RC-L1s using logistic regression adjusted for sex, age, and family history of PD did not identify any individual RC-L1s significantly associated with PD in the PPMI cohort after correction for multiple testing (Tables S1 and S2). The polymorphic nature of these elements means that each individual will have a different complement of RC-L1s and the total number present in the genome will differ. Therefore, across the 70 polymorphic RC-L1 loci (two on the X chromosome excluded), we determined the total number of alleles with an RC-L1 present in each individual’s genome. The two RC-L1s on the X chromosome were excluded to enable male and female subjects to be analyzed together to investigate the effects of the burden of RC-L1s. In the healthy controls, the total number of RC-L1s present at these 70 loci ranged from 29–51 with a mean of 40 and in comparison, the PD subjects had a range of 32–53 with a mean of 41 (Figure S1). Using linear regression adjusted for gender, age, and family history of disease, there was no significant association of PD with an increasing number of RC-L1s (β = 0.32, *p* = 0.14). Next, the healthy controls and PD subjects were categorized across 12 different groupings to determine if having above a certain number of RC-L1s present at the 70 loci affected the likelihood of having PD. For example, in the first group, those individuals with ≤34 RC-L1s present were compared to those with ≥35 RC-L1s and the point at which the individuals were divided into categories increased by one RC-L1 until the final grouping of ≤45 RC-L1s was compared to those ≥46 RC-L1s (Table S3). This analysis found there was no significant association of PD above a specific number of RC-L1s present (Table S3).

**Figure 3:**
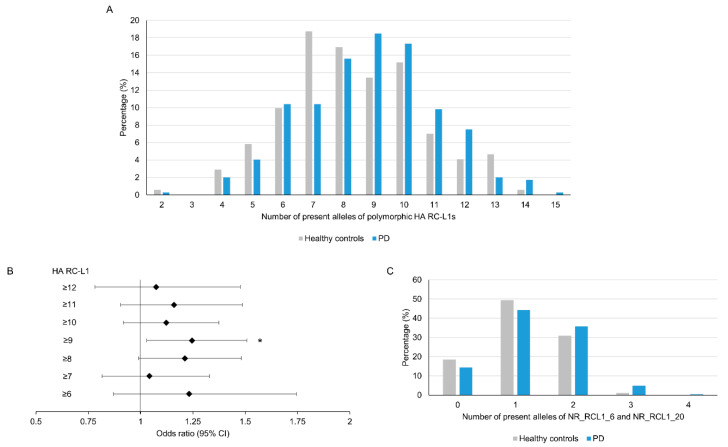
An increase in the number of highly active RC-L1s is significantly associated with Parkinson’s disease. In total, 22 reference and 50 non-reference RC-L1s were identified as polymorphic for their presence and 18 of these were classified as highly active (HA). The total number of alleles with an HA RC-L1 insertion present was calculated for each individual in which 16 of HA RC-L1 genotypes (2 present on X chromosome were excluded) were available. The number of alleles with an HA RC-L1 present ranged from 2 to 14 in the healthy controls and 2 to 15 in PD subjects. (**A**) A comparison of the percentage of healthy controls and PD subjects with defined numbers of HA RC-L1s present. (**B**) The forest plot represents the odds ratio of having PD based on an increasing number of the polymorphic HA RC-L1s being present in an individual’s genome. The likelihood of having PD was significantly associated with having ≥9 of the polymorphic RC-L1s present (OR 1.25, 95% CI 1.03–1.51, *p* = 0.02). The black diamonds represent the odds ratio and black lines the 95% confidence intervals. Healthy controls *n* = 171, PD *n* = 346. * *p* < 0.05 (**C**) Comparison of the percentage of healthy controls and PD subjects with present alleles at NR_RCL1_6 and NR_RCL1_20 loci, which were the only two polymorphic RC-L1s that were in the top quartiles of percentage activity in the cellular retrotransposition assay and the number of germline and somatic 3′ transductions the elements were the source of. Healthy controls *n* = 178, PD *n* = 368.

As outlined in Figure 2, the level of retrotransposition activity of individual RC-L1 varies considerably, with only a small number located at the higher end of each parameter; therefore, we focused our analysis on those highly active (HA) RC-L1s. The HA RC-L1 were defined as those polymorphic elements in the PPMI cohort that were in the top quartile of either percentage activity in the cellular retrotransposition assay (>92.3%) or the number of germline (>3) or somatic (>22) 3′ transductions the elements were the source of. Using this definition, 18 of the 72 polymorphic RC-L1 were defined as HA, with only two RC-L1 (NR_RCL1_6 and NR_RCL1_20) that were HA by all three parameters (Tables S1 and S2). The total number of HA RC-L1s present per individual across 16 loci (two on the X chromosome excluded) was calculated and using linear regression adjusted for gender, age, and family history of PD, a non-significant trend was observed of an increased risk of PD with an increasing number of HA RC-L1s present (β = 0.18, *p* = 0.09). The percentage of healthy controls and PD subjects with different numbers of HA RC-L1s present is shown in Figure 3A. To determine if having above a certain number of HA RC-L1s was associated with disease, the healthy controls and PD subjects were categorized incrementally based on the total number of HA RC-L1s present across seven groupings from those with ≥6 up to ≥12 (Table S4). Logistic regression adjusted for gender, age, and family history of PD was performed for these seven groupings, which identified an association of PD with ≥9 HA RC-L1 present (OR 1.25, 95% CI 1.03–1.51, *p* = 0.02) (Figure 3B and Table S4). In the healthy controls, 45% of individuals had ≥9 HA RC-L1 in comparison to the 57.2% of PD subjects. Analysis of the only two HA RC-L1 that were in the top quartile of all three parameters (NR_RCL1_6 and NR_RCL1_20) identified a significant increase, although small, in PD risk as the number of present alleles at these two loci increased (β = 0.08, *p* = 0.04) and the distribution of the number of HA RC-L1s present in healthy controls and PD subjects is shown in Figure 3C.

### 2.3. Individuals with a Higher Number of Highly Active RC-L1s Show an Increase in Markers of Parkinson’s Disease Progression

**Figure 4:**
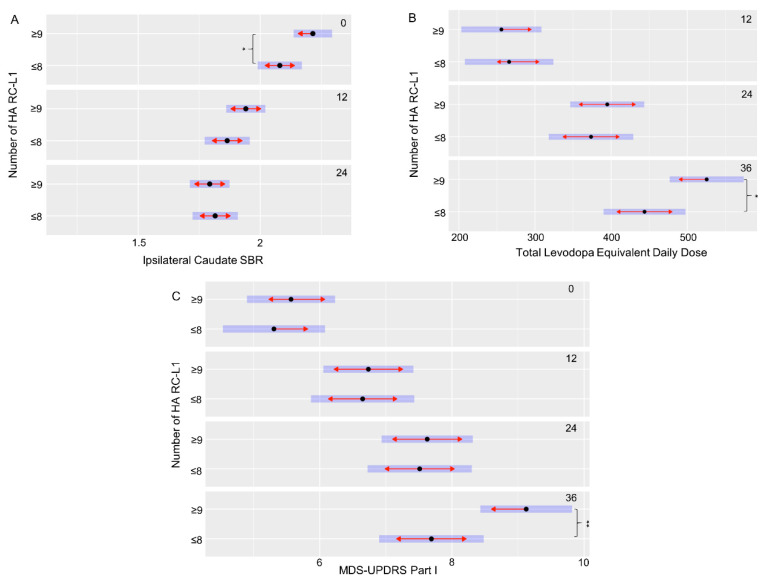
An increased burden of HA RC-L1s across the genome is associated with a significant increase in markers of Parkinson’s disease progression. (**A**) PD subjects with ≥9 RC-L1s had a significantly lower ipsilateral caudate SBR than those with ≤8 RC-L1s at baseline (0 months) (*p* = 0.03). (**B**) PD subjects with ≥9 RC-L1s had a significantly higher mean total levodopa equivalent daily dose than those with ≤8 RC-L1s at 36 months (*p* = 0.03). (**C**) PD subjects with ≥9 RC-L1s had a significantly higher mean MDS-UPDRS part I score than those with ≤8 RC-L1s at 36 months (*p* = 0.008). Black dots represent the mean, the blue bars are the 95% confidence intervals and red arrows are the comparison arrows between the means. * *p* < 0.05 and ** *p* < 0.01.

The PPMI cohort had extensive clinical and phenotypic data collected at multiple time points (0, 12, 24, and 36 months) to measure changes over the disease course. This includes scales to assess symptoms of PD, such as the Movement Disorder Society—Unified Parkinson’s Disease Rating Scale (MDS-UPDRS), Scales for Outcomes in Parkinson’s disease—autonomic dysfunction (SCOPA-AUT) cognitive assessments, and DaTscan imaging. Using a linear mixed-effects model to compare the clinical and phenotypic data of the PD subjects with ≤8 HA RC-L1s to those with ≥9, 11 features that were significantly different at specific time points of the disease were identified (Table 2). However, seven of those features were all related to a significant difference at baseline (0 months) between the striatal bindings ratios (SBRs), a measure by DaTscan imaging of the binding of a radioligand to the dopamine transporter (DAT) present on the presynaptic membrane of nigrostriatal neurons, in the caudate and striatum. A lower SBR represents a lower level of the DAT present and therefore a greater degree of degeneration of dopaminergic neurons. These measurements are interrelated, and all seven features showed the same direction in the relationship between the categories of HA RC-L1 the subjects belonged to and their SBR. The individuals with ≥9 HA RC-L1s had a significantly higher mean SBR in all of the seven features at baseline than those with ≤8 (Table 2). However, at both 12 and 24 months (the latest time point at which SBR data was available), there was no longer a significant difference between those PD subjects with ≥9 HA RC-L1s compared to those with ≤8, demonstrating a higher rate of DAT loss in the subjects with ≥9 HA RC-L1s in the 24-month period. Figure 4A illustrates the significant difference in the ipsilateral caudate SBRs between PD subjects at baseline with ≥9 HA RC-L1s (2.22, 95% CI 2.14–2.29) and those with ≤8 (2.08, 95% CI 1.99–2.17) (*p* = 0.03). The reduction in the SBR of the ipsilateral caudate on average is higher in those individuals with ≥9 HA RC-L1s (0.28 from 0–12 months and 0.15 from 12–24 months) compared to individuals with ≤8 (0.22 from 0–12 months and 0.04 from 12–24 months).

Three of the other significantly different associated features in this analysis of the PPMI data were with scores used to measure different aspects of disease progression (MDS-UPDRS part I, SCOPA-AUT gastrointestinal, and Montreal Cognitive Assessment (MoCA)) (Table 2). For example, the MDS-UPDRS is used to measure non-motor and motor symptoms of PD (Part I-non-motor aspects of experiences of daily living, Part II-motor aspects of experiences of daily living, Part III-motor examination, and the total score is the combined data from part I–III) and higher scores are associated with increasing disease severity. The PD subjects with ≥9 HA RC-L1 had a significantly higher mean MDS-UPDRS part I score (9.12, 95% CI 8.43–9.82) at 36 months compared to those with ≤8 HA RC-L1s (7.69, 95% CI 6.90–8.48) (*p* = 0.008) (Figure 4C). The final significantly associated feature we defined was a higher mean total levodopa equivalent daily dose at 36 months (525, 95% CI 477–574) in PD subjects with ≥9 HA RC-L1s compared to those with ≤8 (444, 95% CI 390–498) (*p* = 0.03) (Figure 4B).

## 3. Discussion

The combination of an individual’s genetics and their environment will determine the risk of developing Parkinson’s disease. Highly penetrant variants in several genes cause monogenic forms of PD; however, the majority of cases are sporadic but still have a genetic component. Large-scale genome-wide association studies have identified 90 single nucleotide polymorphisms associated with sporadic PD [31]; however, a large proportion of the genetic variants contributing to PD risk have yet to be identified [32]. Therefore, we evaluated a type of genetic element, RC-L1s, yet to be addressed genome wide for their role in the predisposition to and progression of PD. Using WGS data from the PPMI cohort, we identified 22 reference and 50 non-reference RC-L1 loci that were polymorphic for their presence or absence, and analysis of these elements did not identify any individual RC-L1 loci associated with PD. However, focusing on the burden of HA RC-L1s, we demonstrated that above a specific threshold of HA RC-L1s (≥9) per genome was associated with PD (OR 1.25, 95% CI 1.03–1.51, *p* = 0.02) (Figure 3). This finding was specific to those elements demonstrating the greatest retrotransposition activity and not to RC-L1 loci in general, suggesting the functional and biological properties of these elements are important factors in understanding their role in PD.

In addition to the significantly increased risk of PD associated with a higher number of HA RC-L1s, we also identified significant differences in markers of disease progression between individuals with ≥9 HA RC-L1s present compared to those individuals ≤8. DaTscans are a specific type of single-photon emission computed tomography imaging used to visualize the levels of DAT in the striatum as a measure of the loss of nigrostriatal neurons and is used as a diagnostic tool for PD [33]. The degeneration of dopaminergic neuron projections into the striatum from the substantia nigra results in a reduction of the DAT and therefore lower SBRs [34]. DaTscans at repeated time points are also a useful measure of the progression of the disease. PD subjects with ≥9 HA RC-L1s present had significantly higher SBRs in the striatum at baseline than those with ≤8, indicating a lower level of dopaminergic neuron degeneration in those individuals with ≥9 (Table 2). However, at 12 and 24 months, there was no longer a significant difference between the two groups, suggesting a more rapid loss of dopaminergic neurons during this period of time in PD subjects with ≥9 HA RC-L1. Analysis of DaTscan data in the future beyond 24 months of follow-up will be important to determine if the rate of DAT loss is similar between the two groups of PD subjects or if the individuals with ≥9 HA RC-L1s continue to exhibit a higher rate of loss. An indication that individuals with ≥9 HA RC-L1s may continue to progress faster is that at 36 months, but not at 12 and 24 months, they require a significantly higher total levodopa equivalent daily dose compared to those with ≤8 (Figure 4B). In addition, the MDS-UPDRS part I score, a marker of disease progression, is significantly higher at 36 months in those with ≥9 HA RC-L1s (Figure 4C).

In our study, we demonstrated in the PPMI cohort that above a certain threshold of HA RC-L1s, the risk of PD is increased and is associated with progression of the disease. The burden of HA RC-L1s could not only be an initiator of dopaminergic degeneration but also exacerbate it. In both a cell line model using human dopaminergic LUHMES cells and a mouse model of PD, L1s were activated in response to mitochondrial distress [19] and mitochondrial dysfunction is one the prominent molecular pathways involved in PD [35]. In another study utilizing a mouse model (Engrailed-1 heterozygotes), midbrain dopaminergic neurons degenerate, which coincides with an increase in L1 expression leading to DNA strand breaks, suggesting the protein Engrailed-1 in the adult represses L1s to protect these neurons [20]. We hypothesize that the activation of L1s in response to cellular stress and/or ageing in dopaminergic neurons would be more detrimental in individuals with a higher number of HA RC-L1s. The RC-L1s may not be a trigger of neurodegeneration but contribute to the process and increase the stress the cells undergo. The expression of RC-L1 mRNA and encoded proteins may contribute to cellular dysfunction through several mechanisms, including DNA damage from the endonuclease activity of ORF2p, somatic insertions disrupting gene function, and the triggering of an immune response [36,37]. If a mechanism of action can be firmly established for these elements in PD, this represents a potential novel therapeutic target for slowing progression and drugs, such as reverse transcriptase inhibitors, could be used that are known to inhibit L1 mobilization [38,39].

## 4. Materials and Methods

### 4.1. Identification of Retrotransposition Competent L1s

RC-L1s were defined as those elements that either demonstrated activity in a cellular retrotransposition assay, were a source element for insertions in the germline, in tumors, or may have fulfilled more than one of the three criteria [22,23,24,25,29,30]. The genomic coordinates for the L1s analyzed for their activity in a cellular retrotransposition assay by Brouha et al. [22] were identified through either the primer sequences reported in the supplementary data or through the sequence fasta files available in the NCBI database (https://www.ncbi.nlm.nih.gov/). For those L1s analyzed by Beck et al. [23] for activity in a cellular retrotransposition assay, the sequence provided in the supplementary data was searched using the Blat tool from the UCSC genome browser (https://genome.ucsc.edu/) to identify the genomic coordinates of the insertion. Genomic coordinates for those L1s that were reported as source elements for 3′ transduction events in the germline or tumors were obtained from the supplementary data of the following publications [25,29,30]. Allele frequency data was collated for the RC-L1s that it was available for [22,23,25,26]. All genomic coordinates were converted to hg38 using the liftover tool from the UCSC genome browser. The reference and non-reference RC-L1s were separated into two lists in Tables S1 and S2, respectively.

### 4.2. Genotyping of RC-L1s in Whole-Genome Sequencing Data from the PPMI Cohort

Whole-genome sequencing data in bam format (aligned to hg38) was obtained from the PPMI cohort (www.ppmi-info.org/data). Reference and non-reference L1s were genotyped in 372 PD subjects and 178 healthy controls using the structural variant caller Delly2 (https://github.com/dellytools/delly) [40] and Mobile Element Locator Tool (MELT) [25], respectively. Reference retrotransposons that are polymorphic for their presence or absence are called deletions relative to the reference genome; therefore, the coordinates of the structural variants classified as deletions by Delly2 that overlapped with the reference RC-L1 (Table S1) were extracted. The variants were inspected manually using UCSC genome browser to ensure they corresponded to L1s and start and end coordinates were consistent with those reference RC-L1s in Table S1. Non-reference L1s were genotyped using MELT with default parameters and the L1s corresponding to non-reference RC-L1s in Table S2 were extracted for further analysis.

### 4.3. Statistical Analysis of Polymorphic RC-L1s

There were 22 reference and 50 non-reference RC-L1s identified as polymorphic in the PPMI cohort. Logistic regression adjusted for sex, age, and family history of PD was performed for the 72 polymorphic RC-L1s and *p* values were corrected for multiple testing (Bonferroni) using PLINK (v1.07) [41]. Highly active (HA) RC-L1s were defined as belonging to the top quartile of percentage activity in the cellular retrotransposition assay or the number of either germline or somatic 3′ transductions of the 72 polymorphic RC-L1s. In total, 18 of the 72 RC-L1s were classified as HA. The total number of present alleles for the 70 polymorphic RC-L1 loci and the 16 HA RC-L1 loci located on the autosomes (two excluded on the X chromosome so males and females could be analyzed together) was calculated for each individual. Linear regression was performed adjusted for gender, age, and family history of PD to analyze the association of either the total number of RC-L1s or HA RC-L1s present and disease status. In addition, both healthy controls and PD subjects were categorized based on either the number of alleles with an RC-L1 or an HA RC-L1 present to analyze the association between the likelihood of having PD above a certain number of either RC-L1s or HA RC-L1s. These groupings were chosen to ensure when comparisons between the two categories were performed as the smallest category did not contain fewer than 5% of either the healthy controls or PD subjects. Logistic regression adjusted for gender, age, and family history of PD was performed on these RC-L1 or HA RC-L1 groupings to determine if above a number of these defined elements present was associated with PD.

### 4.4. Association Analysis of the Burden of Highly Active RC-L1s with Clinical Features and Progression Markers of PD in PPMI Longitudinal Data

Clinical and phenotype data collected at four time points (0, 12, 24, and 36 months) for the PPMI cohort were downloaded from www.ppmi-info.org/data. In order to analyze the burden of HA RC-L1s in relation to the progression of PD, we applied linear mixed-effect modelling with the clinical features and the follow-up times as fixed-effect factors and patient as a random factor. The statistically significant features were further used for the pairwise comparisons (between HA RC-L1 groups) as a function of visits. We applied estimated marginal means to calculate the mean response of each factor and performed pairwise comparison between different HA RC-L1 groups for all visits separately to identify the change in means during the progression of the PD. The R packages *lmerTest* (https://github.com/runehaubo/lmerTestR) and *emmeans* (https://github.com/rvlenth/emmeans) were used.

## Figures and Tables

**Table 1:** Demographics of the Parkinson’s Progression Markers Initiative cohort. The healthy controls and Parkinson’s disease (PD) subjects were divided into two groups those with ≤8 and those with ≥9 of the RC-L1 present that were identified as polymorphic and highly active (HA). Genotypes were not available at all 16 RC-L1 loci for all individuals; therefore, the total number of combined individuals from the ≤8 and ≥9 HA RC-L1 groups is lower than the cohort total. * One PD subject with family history data missing.

Variable	Healthy Controls			PD Subjects		
	Total (*n* = 178)	≤8 HA RC-L1 (*n* = 94)	≥9 HA RC-L1 (*n* = 77)	Total (*n* = 372)	≤8 HA RC-L1 (*n* = 148)	≥9 HA RC-L1 (*n* = 198)
Gender						
Male	116 (65%)	61 (65%)	49 (64%)	242 (65%)	98 (66%)	128 (65%)
Female	62 (35%)	33 (35%)	28 (36%)	130 (35%)	50 (34%)	70 (35%)
Age						
Mean (min, max)	61.3 (30.6–82.7)	60.8 (31.2–79.3)	61.4 (30.6–82.7)	61.9 (33.5–84.9)	62.9 (33.5–83.0)	61.9 (39.2–84.9)
Age of onset						
Mean (min, max)	na	na	na	60.0 (29.2–83.0)	60.9 (29.2–81.3)	60.0 (35.9–83.0)
Family history of PD *						
1st degree family member	0 (0%)	0 (0%)	0 (0%)	51 (15.7%)	16 (10.8%)	29 (14.7%)
Other family member	10 (5.6%)	6 (6.4%)	4 (5.2%)	43 (11.6%)	16 (10.8%)	24 (12.2%)
No family member	168 (94.4%)	88 (93.6%)	73 (94.8%)	277 (74.7%)	116 (78.4%)	144 (73.1%)

**Table 2:** Clinical features of Parkinson’s disease that were significantly associated with different numbers of HA RC-L1. The mean and 95% confidence intervals (CI) are reported for each feature that were significantly different between PD subjects with ≤8 HA RC-L1s compared to those with ≥9.

Associated Feature	Time of Visit (Months)	≤8 HA RC-L1 Mean (95% CI)	≥9 HA RC-L1 Mean (95% CI)	*p*-Value
MDS-UPDRS Part I Score	0	5.31 (4.53–60.8)	5.57 (4.90–6.23)	0.62
	12	6.65 (5.87–7.43)	6.74 (6.06–7.42)	0.87
	24	7.51 (6.72–8.30)	7.63 (6.94–8.32)	0.83
	36	7.69 (6.90–8.48)	9.12 (8.43–9.82)	**0.008**
Total Levodopa Equivalent Daily Dose	12	266 (208–324)	256 (203–308)	0.80
	24	374 (318–429)	395 (346–443)	0.57
	36	444 (390–498)	525 (477–574)	**0.03**
SCOPA-AUT Gastrointestinal Score	0	2.25 (1.88–2.62)	1.96 (1.64–2.27)	0.23
	12	2.80 (2.43–3.17)	2.83 (2.50–3.15)	0.91
	24	2.87 (2.49–3.24)	2.94 (2.61–3.37)	0.78
	36	2.85 (2.48–3.23)	3.49 (3.16–3.82)	**0.01**
MoCA Score (adjusted for education)	0	27.6 (27.2–28.0)	27.0 (26.6–27.4)	**0.04**
	12	26.4 (25.9–26.8)	26.6 (26.2–27.0)	0.48
	24	26.5 (26.0–26.9)	26.6 (26.2–27.0)	0.56
	36	26.4 (26.0–26.9)	26.6 (26.2–27.0)	0.45
Highest caudate measure (SBR)	0	2.10 (2.01–2.19)	2.24 (2.16–2.32)	**0.02**
	12	1.89 (1.80–1.98)	1.96 (1.88–2.04)	0.26
	24	1.83 (1.74–1.92)	1.82 (1.74–1.90)	0.84
Mean caudate measure (SBR)	0	1.93 (1.84–2.01)	2.05 (1.98–2.12)	**0.03**
	12	1.74 (1.66–1.83)	1.79 (1.72–1.86)	0.40
	24	1.67 (1.58–1.75)	1.66 (1.58–1.73)	0.88
Ipsilateral caudate measure (SBR)	0	2.08 (1.99–2.17)	2.22 (2.14–2.29)	**0.03**
	12	1.86 (1.77–1.96)	1.94 (1.86–2.02)	0.22
	24	1.82 (1.72–1.91)	1.79 (1.71–1.87)	0.72
Contralateral caudate measure (SBR)	0	1.78 (1.69–1.86)	1.89 (1.82–1.96)	**0.045**
	12	1.62 (1.54–1.70)	1.64 (1.57–1.71)	0.74
	24	1.52 (1.43–1.60)	1.52 (1.45–1.59)	0.95
Highest striatum measure (SBR)	0	3.03 (2.90–3.17)	3.24 (3.12–3.35)	**0.02**
	12	2.67 (2.53–2.80)	2.76 (2.64–2.88)	0.33
	24	2.56 (2.42–2.70)	2.55 (2.43–2.67)	0.94
Mean striatum measure (SBR)	0	1.36 (1.30–1.42)	1.44 (1.40–1.50)	**0.04**
	12	1.21 (1.15–1.27)	1.24 (1.19–1.30)	0.43
	24	1.15 (1.09–1.22)	1.15 (1.10–1.21)	0.97
Ipsilateral striatum measure (SBR)	0	3.00 (2.87–3.14)	3.20 (3.09–3.32)	**0.03**
	12	2.63 (2.49–2.77)	2.73 (2.61–2.85)	0.25
	24	2.54 (2.40–2.68)	2.52 (2.40–2.64)	0.84

The *p* values are in bold to highlight those that are significant (*p* < 0.05).

## Supplementary Materials

Supplementary materials can be found at www.mdpi.com/xxx/s1.Table S1: Compiled list of reference RC-L1s. Table S2: Compiled list of non-reference RC-L1s. Table S3: Results for logistic regression analysis of the 12 RC-L1 groupings. Table S4: Results for logistic regression analysis of the 7 highly active RC-L1 groupings. Figure S1: Comparison of the percentage healthy controls and PD subjects with defined numbers of RC-L1s.

## Abbreviations

DAT	Dopamine Transporter
CI	Confidence Interval
HA	Highly Active
LINE-1/L1	Long interspersed element-1
MDS-UPDRS	Movement Disorder Society—Unified Parkinson’s Disease Rating Scale
MOCA	Montreal Cognitive Assessment
NR_RCL1	Non-reference retrotransposition competent long interspersed element-1
ORF	Open reading frame
PD	Parkinson’s disease
PPMI	Parkinson’s progression markers initiative
RC	Retrotransposition competent
Ref_RCL1	Reference retrotransposition competent long interspersed element-1
SBR	Striatal binding ratio
SCOPA-AUT	Scales for Outcomes in Parkinson’s disease—autonomic dysfunction
TSD	Target site duplication
UTR	Untranslated region
