# Supplementary figures and images for: An Increased Burden of Highly Active Retrotransposition Competent L1s Is Associated with Parkinson’s Disease Risk and Progression in the PPMI Cohort
Source: Int J Mol Sci. 2020 Sep 8;21(18):6562. doi: 10.3390/ijms21186562 (PMC7554759; doi:10.3390/ijms21186562)

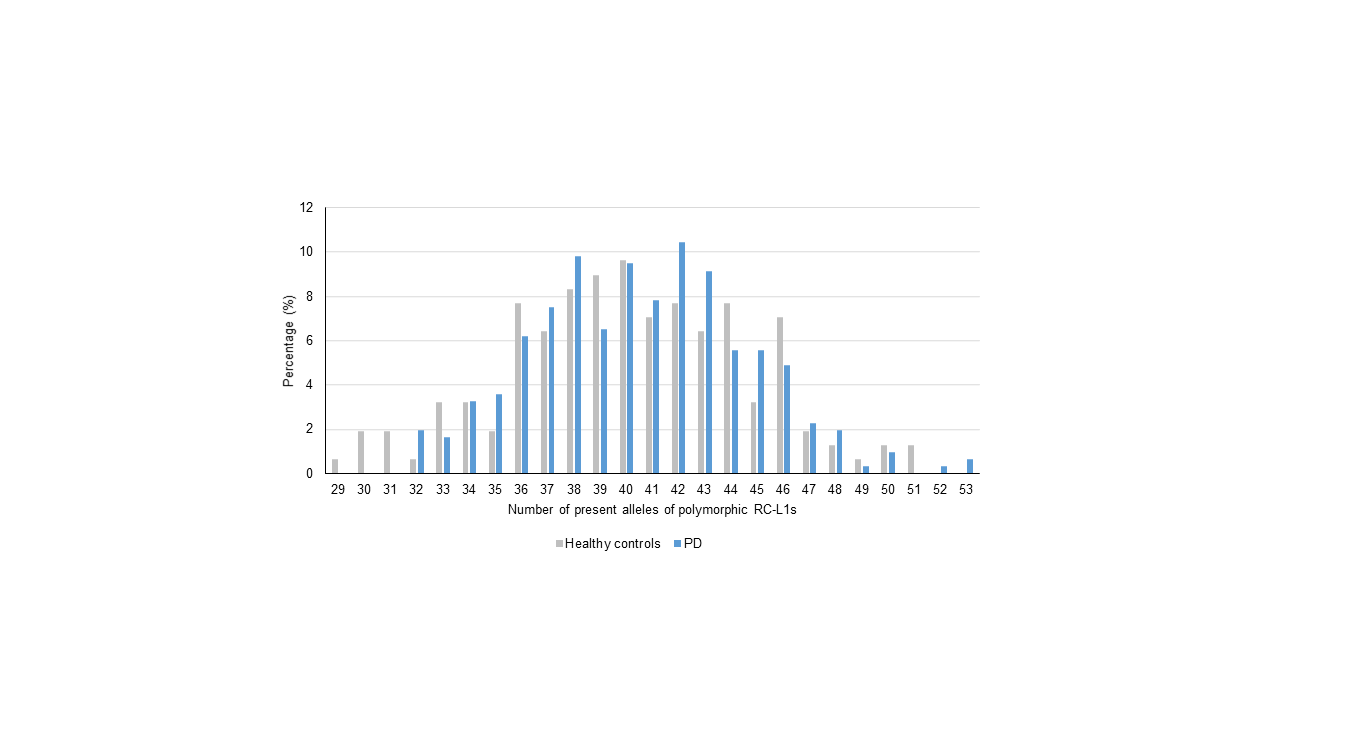

Supplement: Supplementary file 1 [file ijms-21-06562-s001.zip › supp data/Supplementary Figure 1.tif]
